# Molecular detection of field predation among larvae of two ladybird beetles is partially predicted from laboratory experiments

**DOI:** 10.1038/s41598-018-20830-2

**Published:** 2018-02-07

**Authors:** Gabriele Rondoni, Saleh Fenjan, Valeria Bertoldi, Fulvio Ielo, Khaled Djelouah, Chiaraluce Moretti, Roberto Buonaurio, Carlo Ricci, Eric Conti

**Affiliations:** 1 Department of Agricultural, Food and Environmental Sciences, University of Perugia, Via Borgo XX Giugno 74, 06121 Perugia, PG Italy; 2 CIHEAM, Mediterranean Agronomic Institute, Via Ceglie 9, 70010 Valenzano, BA Italy

## Abstract

Despite the fact that natural enemies can synergistically contribute to herbivore pest suppression, sometimes predators engage in intraguild predation (IGP) that might dampen trophic cascades. DNA-based gut-content analysis has become common in assessing trophic connections and biocontrol potential by predators in field systems. Here, we developed a molecular technique that can be used to unravel predation among two ladybirds, *Coccinella septempunctata* and *Hippodamia variegata*, and their shared prey, *Aphis gossypii*. Both ladybirds may provide effective control of the pest. Therefore, understanding their likelihood to engage in IGP is crucial for conservation biological control. Ladybird specimens were collected in melon crop. DNA extraction, primer design and evaluation were conducted. Detectability of prey DNA did not differ significantly between the two ladybirds. *H*. *variegata* exhibited higher predation on *A*. *gossypii* than *C*. *septempunctata* (90.6% vs. 70.9%) and data correction based on DNA detectability confirmed this ranking. IGP was similar among the two species, although corrected data might suggest a stronger predation by *C*. *septempunctata*. Intriguingly, IGP by *C*. *septempunctata* was lower than predicted by laboratory bioassays, possibly due to the high complexity that arises under field conditions. Implications of our results for biological control and perspectives for ecological network analysis are discussed.

## Introduction

In agricultural systems, biological control of herbivore pests by natural enemies provides a valuable resource for the economy, which has been estimated as $ 4.49 billion annually only in the United States^1^. In this respect, evidence supports the existence of a positive relationship between predator biodiversity and biocontrol efficacy^2–4^. In general, natural enemies can synergistically contribute to increasing herbivore control (reviewed by ref.^5^) due to proposed mechanisms of niche complementarity or facilitation (reviewed by refs^6,7^). However, besides this broad scenario of the beneficial effects of biodiversity on pest control, adding more species in the system sometimes has no effect or may even result in unfavourable herbivore population increase due to the weakening of trophic cascades^8^. This is true, especially when dealing with generalist predators, which may feed not only on herbivores but also upon alternative preys and even conspecific or heterospecific predators (reviewed by ref.^6^). This interaction is known as intraguild predation (IGP) and takes place when a predator species (IG-predator) consumes another predator (IG-prey), both species sharing a common prey^9^.

IGP has received great attention in recent years especially because of its negative effect on local biodiversity. For example, an exotic predatory ladybird species that establishes in a new area might gain an advantage by IGP upon members of the invaded community. As a consequence, native species are often dislodged from their usual habitats (reviewed by refs^10–12^). In addition, IGP can result in a negative effect on pest suppression by altering the efficacy of natural enemies, thus weakening trophic cascades^13^. Several studies have highlighted the complexity and impact of IGP on arthropod communities^8^, with positive or negative effects depending on the system under investigation.

The occurrence and ecological consequences of IGP have been studied mostly under laboratory conditions through Petri dish assays^11,14–16^. IGP studies based on long-term experiments and conducted in complex ecosystems are few, because traditional methods are not appropriate to detect and quantify such interactions. In fact, quantification of predatory events under field conditions is difficult, although a few studies have been attempted, specifically by using video recording systems^17^.

Recently, the development of molecular techniques has made the detection of predation between arthropods relatively easy (reviewed by refs^18–20^). The application of this technique to IGP research can provide a better understanding of the predator-prey interactions and the effect on the biological control of herbivore pests. As an example, it was shown that although generalist predators, such as carabid beetles, may provide temporary control of aphids, they also exhibit coincidental IGP upon aphid parasitoids, ultimately resulting in the reduction of biological control on a long-term prospect^21^. Concerning ladybirds, molecular analysis of predation has been applied to detect the occurrence of IGP events in the field^22–27^.

Open field crops, including horticultural ones, are characterised by fluctuations in predator and prey densities over space and time. In this conditions, to sustain their food web, generalist or stenophagous predators rely on selected herbivore pests, but also they regularly switch upon alternative food sources, including intraguild preys^11,21,28,29^. Considering that the entity of IGP is still uncertain, there is urgent need to develop molecular techniques to be used for unravelling trophic food webs in agricultural ecosystems.

Here, we developed and applied new primers for PCR analysis to evaluate aphid predation and IGP by coccinellid species in melon, *Cucumis melo* L., fields. Melon is an economically important crop that can be attacked by several insect pests, including the cotton-melon aphid, *Aphis gossypii* Glover^30^. This aphid is very widespread, as it belongs to the 1% of the alien insect species that are present in more than 40 Countries, and it is the most economically important pest of melon crop^31^. Ladybirds, mostly *Coccinella septempunctata* L. and *Hippodamia variegata* (Goeze) (Coleoptera: Coccinellidae), are considered important and efficient naturally occurring predators of *A*. *gossypii*^32^, but since they are euryphagous in their diet breadth, it is predicted that they are likely to engage in IGP^33^.

In addition, we aimed at developing a method that allows comparing IGP levels obtained under laboratory conditions (row data from ref.^33^) with results from field-collected larvae. Molecular data typically consist in several positive or negative outcomes and it is not easy to straightforwardly derive ecological significance^28^. Hence, statistical procedures are needed to interpret raw qualitative data obtained from molecular analysis^34^. Monte Carlo simulations have been previously used to derive predation and confidence intervals when random predation is expected (reviewed by ref.^28^). Furthermore, Welch, *et al*.^35^ used parametric bootstrap and Bayesian inference as a first step towards the quantitative evaluation of predation rates obtained from the gut-content analysis. Here, using a similar procedure, we attempted a statistical comparison of frequencies observed under natural conditions with frequencies obtained from simulated databases in which the predicted predation is derived from laboratory contexts. The proposed comparison is relevant in particular for predatory ladybird beetles, where the huge literature existing on the likelihood of IGP under laboratory conditions might be used to predict IGP under the real conditions that arise in the agroecosystems.

## Results

**Figure 1:**
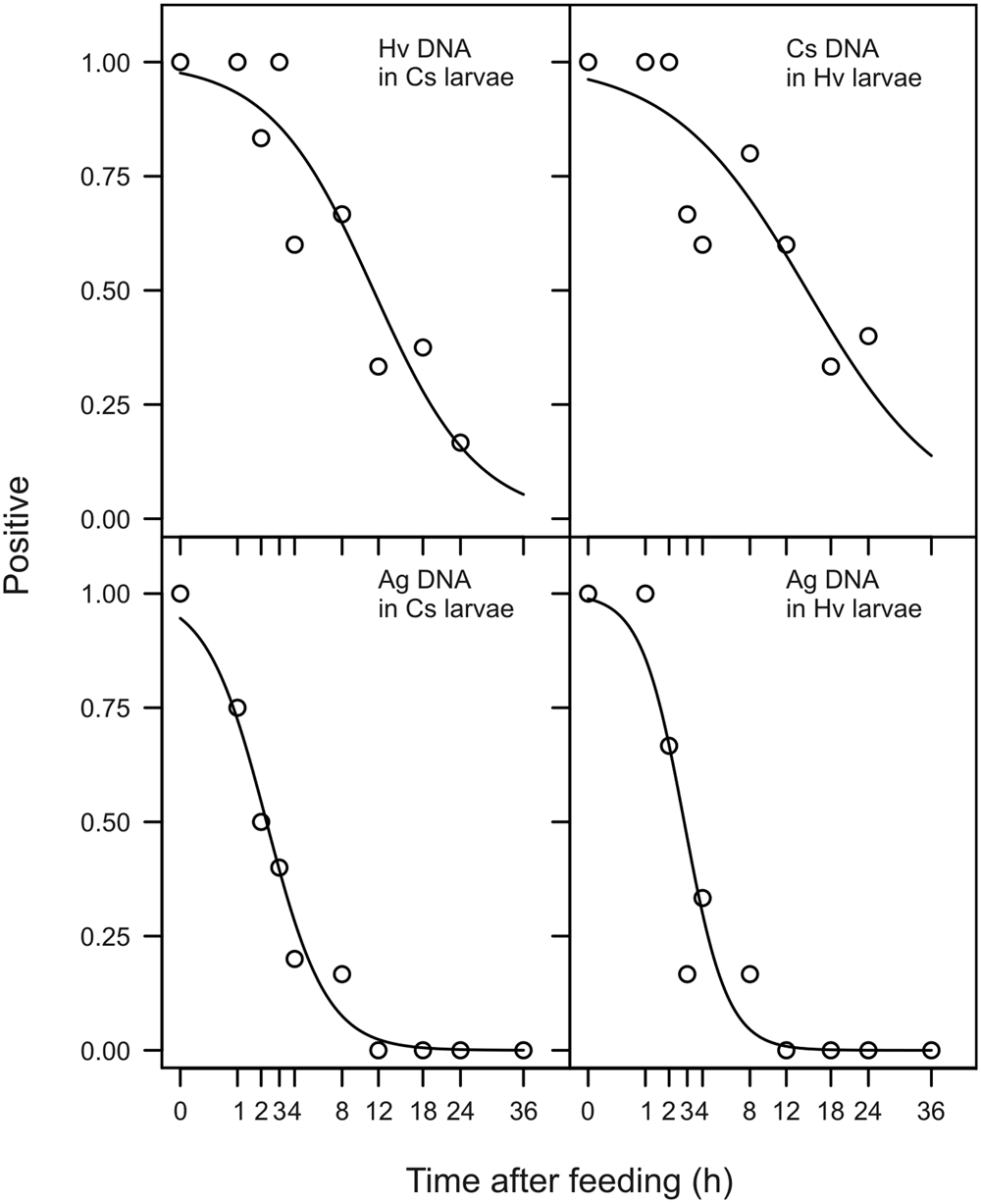
Positive detection of prey DNA following consumption by predatory 4^th^ instars. Hv = *H*. *variegata*; Cs = *C*. *septempunctata*; Ag = *Aphis gossypii*. Symbols represent observed proportions of positive amplifications. Lines represent fitted binomial GLMs.

Based on *Cytochrome Oxidase I* (*COI*) mitochondrial region, primers were designed for *H*. *variegata*, *C*. *septempunctata* and *A*. *gossypii* (Table 1), which amplify small fragments of 104, 108 and 263 bp, respectively. The designed primers showed to be specific for the three species among several non-target arthropod species. Primer sensitivity was similar between the different types (ladybird or aphid) of target prey. The amount of 156 DS template copies was necessary to amplify *H*. *variegata* and *C*. *septempunctata* DNA and 312 DS template copies to amplify *A*. *gossypii* DNA. The detectability of prey DNA following consumption by predator decreased with the time post feeding (Fig. 1). The half-detectability time (T50) in the gut of *C*. *septempunctata* was 11.40 h and 2.27 h for the detection of *H*. *variegata* and *A*. *gossypii* respectively, while the T50 in the gut of *H*. *variegata* was 14.64 h and 2.81 h for the detection of *C*. *septempunctata* and *A*. *gossypii* respectively. More specifically, the detectability did not differ significantly between the two predators neither in the case of predation upon aphids (LRT: P = 0.55), nor upon ladybirds (LRT: P = 0.58).

**Table 1:** Details of primer sequences designed on the *Cytochrome Oxidase I* (*COI*) mitochondrial region to detect DNA of *Hippodamia variegata*, *Coccinella septempunctata* and *Aphis gossypii*, and expected amplicon sizes.

Insect species	Region	Sequences	Amplicon size
*Hippodamia variegata*	*CO1*	F: 5′-CTGATATAGCATTCCCTCGTCTT-3′	104 bp
		R: 5′-GTTCCAGCCCCTATTTCAACA-3′	
*Coccinella septempunctata*	*CO1*	F: 5′-CCCACCTGCCTTAACCTTACTT-3′	108 bp
		R: 5′-GGCCCATTATGAGCTAAGTTAGAG-3′	
*Aphis gossypii*	*CO1*	F: 5′-GGTATTTGATCAGGTATAATTGGT-3′	263 bp
		R: 5′-ATTAATGAGGGTGGTAATAATCAG-3′

**Figure 2:**
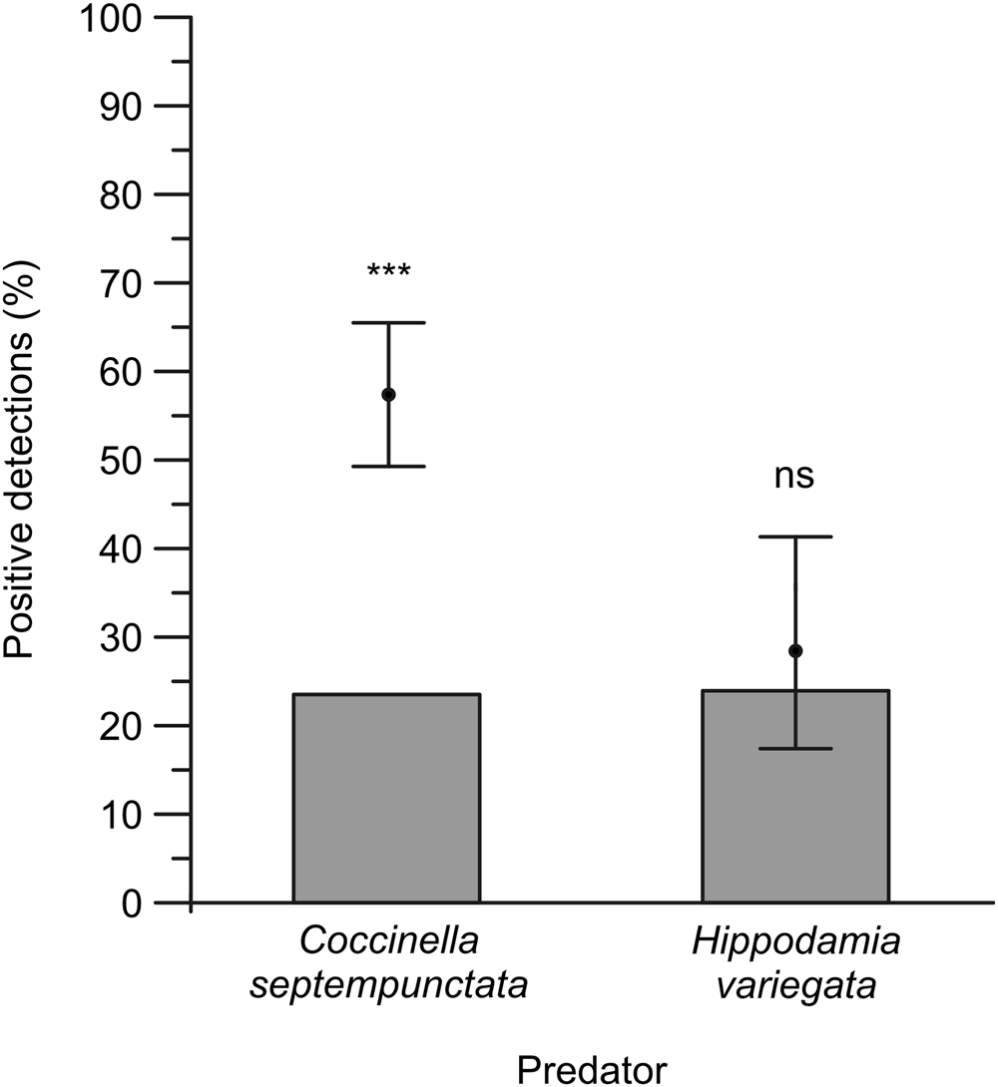
Percentage of *C*. *septempunctata* and *H*. *variegata* 3^rd^ and 4^th^ instars revealed positive for IGP (grey bars) compared with expected predation levels (black dots) and 95% confidence intervals (vertical bars). Comparisons of observed vs. expected predation levels are indicated (*** : P < 0.001, ns : P > 0.05).

In order to evaluate the aphid predation and IGP among the two target coccinellid species, the designed primers were used to detect aphid and coccinellid DNA in the gut content of the field-collected predators (151 *C*. *septempunctata* and 53 *H*. *variegata*). Overall, *C*. *septempunctata* larvae exhibited a lower predation upon aphids if compared with *H*. *variegata* (70.9% vs. 90.6%, binomial GLMM, P < 0.05). Correcting the raw data based on the DNA detectability reduced the differences in aphid predation, but left unchanged the ranking between the two ladybird species (*C*. *septempunctata*: 70.9%; *H*. *variegata*: 78.6%). Additionally, 25.2% of *C*. *septempunctata* preyed upon *H*. *variegata*, compared to 28.3% of vice-versa (binomial GLMM, P > 0.05). Corrected data changed the ranking (*C*. *septempunctata*: 25.2%; *H*. *variegata* 15.6%). Focusing only on the third and fourth instars, IGP observed for *C*. *septempunctata* was significantly lower (P < 0.001) than expected from simulated datasets based on laboratory experiments, whereas IGP by *H*. *variegata* was not different (P > 0.05) (Fig. 2).

## Discussion

Because ladybird beetles have to deal with aphid colonies, which increase quickly but also quickly disappear from the agroecosystems, these predators are often forced to look for alternative preys to sustain their development^36^. In this scenario, some ladybird species might exhibit complex and unpredictable responses that confer them a competitive advantage over similar predators^37^. IGP, in particular, can foster the abundance of one ladybird species upon other predators^11^.

Here the primer pairs we developed allowed us to detect high levels of predation by *H*. *variegata* and *C*. *septempunctata* upon the aphid *A*. *gossypii*. More specifically, *H*. *variegata* appeared to predate on *A*. *gossypii* more than *C*. *septempunctata*. Although the two ladybirds exhibited a similar digestion rate, the adjustment of the raw predation to account for the predator-specific prey detectability reduced the differences between the two predators, but it did not change their relative ranking. Therefore the results suggest that *H*. *variegata* might be a superior competitor for the shared prey.

Besides, we detected similar IGP between *C*. *septempunctata* and *H*. *variegata*, although when correcting the raw data it seemed that *C*. *septempunctata* might be a superior IG-predator, apparently confirming the asymmetrical IGP detected in Petri dish experiments^33^. However, when observed vs. expected data were compared, still the field observed IGP by *C*. *septempunctata* was lower than expected considering a predation probability that follows results from laboratory bioassays, suggesting that laboratory experiments may overestimate field IGP.

The usefulness of data adjustments to account for different detectability has been widely recognized^34^, however, when the decay rates between predators do not differ, it is noteworthy to understand whether or not the corrected data might be preferable to the raw data. In fact, feeding trials only partially explain the likelihood of DNA detectability in the field. Additional factors, part of which are difficult to estimate, might be also relevant. These include the amount of prey items differently consumed and the body size of each individual predator (examined by refs^38,39^), the satiation level which is standardized in the laboratory but may change in the field^28^ and environmental factors, especially the temperature (reviewed by ref.^34^). Therefore, to properly understand raw data obtained from qualitative PCR, it would be helpful to investigate not only the digestion rates but also the effect of insect-specific traits and environmental factors and possibly provide a hierarchy of their relevance.

The use of PCR-based methods for studying the trophic interactions among ladybird predators and prey is an emerging field that has grown exponentially^19^. For example, Thomas *et al*.^24^ detected the DNA of native ladybirds within *H*. *axyridis* in the UK, with 3.7–22.7% of *H*. *axyridis* found to have consumed native species over 3-year surveys. Gagnon *et al*.^22^ revealed that 52.9% of sampled ladybirds in North America soybean fields contained in their gut DNA from other ladybirds. In Italy, 7% of *H*. *axyridis* larvae collected from trees contained the DNA of two native ladybirds, despite the high prevalence of aphid predation^26^.

The adoption of molecular approaches to unravel trophic relationships in the wild requires that primers should be sensitive enough to amplify small amounts of digested prey DNA in the predator’s gut. In fact, such detection is most likely to be successful if the genes that are amplified are present in multiple copies (specifically COI), and if target sequences are relatively short^20^. In our case, fragments amplified by the three designed primer pairs were all short sizes (less than 263 bp). Designing a pair of primers that amplify short fragment sizes may increase the detection period, thus allowing relatively long detection over time^18^. When attempting at detecting predation along multiple trophic levels, false positives may arise due to secondary predation^20^ and introduce a bias in the estimate of aphid predation proportions. However, the detectability of *A*. *gossypii* in the gut of the predator rapidly decreases using our primers, therefore this bias should not be a matter of concern for our assay.

Previous studies demonstrated that *C*. *septempunctata* may act as a strong predator of IG coccinellid preys (reviewed by ref.^11^). Under laboratory conditions, *C*. *septempunctata* predated upon *H*. *variegata* at a higher rate than vice-versa (70% vs. 43% overall) and the likelihood of IGP was reduced at higher aphid densities or older juvenile stages of the IG-prey (22% upon fourth instars vs. 74% upon eggs and second instars)^33^. Similarly, *C*. *septempunctata* larvae exhibited asymmetric predation upon *Adalia bipunctata* (L.), both in Petri arena^40^ and in semi-field experiments^41^. Additionally, Snyder *et al*.^16^ reported asymmetric predation under laboratory conditions of *C*. *septempunctata* upon *Hippodamia convergens* Guérin-Méneville and *Coccinella transversoguttata* Brown. In particular, the latter species was dominant in agricultural fields in Eastern Washington and Northern Idaho, but after the arrival of *C*. *septempunctata* it exhibited niche displacement^42^.

A possible explanation for the lower IGP exhibited by *C*. *septempunctata* larvae in our open field surveys compared to laboratory trials is that the ladybird is rather generalist^43^, therefore its larvae can fulfil their food requirement by feeding on alternative IG-prey types other than *H*. *variegata*. Examples of potential alternative IG-preys that were abundantly detected during surveys include hoverflies, predaceous gall midges, heteropteran zoophagous predators or even other *C*. *septempunctata* larvae (unpublished data). Although more sampling should be conducted to better understand the trophic ecology of the predators, an interesting scenario can be drawn. Coexistence of intraguild predator and intraguild prey is possible if the latter is a superior competitor for the shared prey^44^. In this case, enough resources allow sustaining both the intraguild predator and the intraguild prey. In our case, the fact that *H*. *variegata* exploits the shared prey better than *C*. *septempunctata*, might be relevant in fostering the coexistence between the two species, although with a possible overall reduction of pest suppression. Finke and Denno^45^ empirically demonstrated this scenario. The specialist predator *Tytthus vagus* (Knight) alone was able to control planthoppers on *Spartina* cordgrass. However, when more generalist planthoppers’ predators were intentionally introduced into the system, a weakening of trophic cascades was detected because of IGP by predators upon *T*. *vagus*^45^.

Molecular PCR-based analysis are time-consuming since they require rearing experiments, laboratory feeding trials, field collection and screening of predation. Therefore the interactions under investigations are usually only a subset of all the possible interactions that are expected in a given ecosystem^46^. Field studies are therefore limited and cannot provide an exaustive interpretation of all the possible connections that arises in nature. However, our empirical results may represent a unique input for the development of a more ambitious ecological network analysis (ENA) which takes into consideration the ecological community in its broader sense. ENA is a methodology that allows a holistic analysis of all the possible ecological interactions that occur in a given ecosystem^46^. To derive ecological compartments and connections among different trophic levels, ENA uses empirical data when available (generated for example from PCR or from next generation sequencing technologies), or it requires that simple algorithms should be used to describe unknown interactions^47^. Within the ENA framework, our data can possibly be used to investigate the ecological effect of the establishment of the two ladybirds in new areas where they accidentally or intentionally arrived. Starting from the last two decades of the 20^th^ Century, *C*. *septempunctata* and *H*. *variegata* established in several Countries around the World and in some cases they have become dominant species^48,49^. Through simulation, ENA could provide a useful method to understand the strength of IGP at different post-establishment stages of the two ladybirds and to reveal its role in fostering the establishment process.

In the context of biological control, *H*. *variegata* has been proposed as a candidate for augmentative programs against the melon key pest *A*. *gossypii*^50,51^. By developing specific molecular gut content analyses, we were able to evaluate the potential of IGP between *C*. *septempunctata* and *H*. *variegata* when they share *A*. *gossypii* as a common prey. Our results predict possible coexistence of *H*. *variegata* with *C*. *septempunctata* wherever the two ladybirds co-occur. Although preliminary, our results indicate that, if field releases of predators are necessary to enhance biological control of *A*. *gossypii*, a safe choice might be the release of *H*. *variegata*, which will show good efficacy combined with a moderate risk of IGP. In conclusion, our research represents a baseline to predict the likelihood of predation in the field and investigate trophic relationships in coccinellid assemblages and their role in pest suppression.

## Materials and Methods

### Field sampling

The coccinellid samples were collected in July 2013, from a melon agroecosystem (extension: 30 ha). The field was located in the southern part of the Perugia province, in Central Italy (Coordinates of the central area: N 42.972, E 12.399). The field was managed with standard agricultural practices and integrated pest management strategies. The presence of *A*. *gossypii* populations was detected from June to August. Within the field, a total of twenty-eight sampling units, each consisting of 10 m × 1 m (length × wide) melon crop rows, were randomly selected. The density of *A*. *gossypii* was recorded from a sample of 10 leaves randomly collected for each sampling unit. For each unit, active sampling on the vegetation was carried out for 3 min using a motorised aspirator with a filter gauze mounted on the opening. The coccinellid samples were instantly killed with spray ice to avoid regurgitation, and stored in >95% ethanol. The samples were then transferred to the laboratory, identified based on morphological characters^52,53^, and stored at −20 °C until DNA extraction.

### Insect cultures

Cultures of *C*. *septempunctata* and *H*. *variegata* were established in the laboratory from adults collected from melon fields in Central Italy, and both larvae and adults were reared on an *ad libitum* diet of *Aphis fabae* Scopoli. *Aphis gossypii* winged females were collected from the field and colonies were established in the laboratory on *Hibiscus syriacus* L. plants. Insects were reared in a controlled environmental chamber at 25 ± 1 °C, 70% ± 5% RH, and 14 h L: 10 h D photoperiod.

### DNA extraction, PCR analysis, sequencing, and primer design

Total DNA was extracted from specimens of *H*. *variegata*, *C*. *septempunctata*, and *A*. *gossypii*, using QIAGEN DNeasy Blood & Tissue Kit (QIAGEN Inc., Chatsworth, CA, USA) following the manufacturer’s animal tissue protocol. Universal primers LCO1490 and HCO2198^54^ were used in the polymerase chain reaction (PCR) to amplify a region within *COI* region for sequencing. The final volume of 50 μl consisted of 2× KAPA buffer (KAPA Biosystems), 0.4 μM of each primer, 5 μl of BSA and 2 μl of template DNA. PCR reactions were performed in Bio-Rad MyCycler thermal cycler system (Bio-Rad Laboratories Inc., Hercules, CA, USA). The PCR cycling conditions used were: an initial denaturing step of 94 °C for 60 s, followed by 40 cycles of 90 °C for 60 s, 56 °C for 60 s, and 72 °C for 60 s and a final extension of 72 °C for 4 min. PCR products (10 μl) were separated by electrophoresis in 2% agarose gel stained with ethidium bromide. Amplicons of the expected size were sequenced by BMR Genomics (Padova, Italy) and alignment of forward and reverse sequences for each individual was performed using MEGA7 software. Sequences were submitted to GenBank (accession numbers in the Supplementary information Table S1). Multiple sequence alignment was conducted using MUSCLE^55^, by including sequences of the same or closely related species obtained from GenBank database^56^ (Table S1). Primers (Table 1), designed using the Primer3 website^57^, were optimised and tested for cross-reactivity against the other insects used in the laboratory experiments and additional non-target invertebrates, most of them occurring in the field area. PCR reactions (20 μl final volume, including 1 μl DNA) were performed using the same protocol as above described at the following different annealing temperatures: 62 °C for *H*. *variegata*, 64 °C for *C*. *septempunctata*, and 62 °C for *A*. *gossypii*. Sample cross-contamination during collection with aspirator might be avoided if the individuals are readily frozen then preserved in EtOH^58,59^. In addition, before extractions, all larvae were rinsed with 0.1% NaClO solution to avoid possible carry-on of surface contamination (similar to ref.^60^). DNA integrity was evaluated by PCR amplification and electrophoresis of PCR product (10 μl) in 1% agarose gel stained with ethidium bromide. All reactions were run with positive and negative controls to determine reaction success.

### Evaluation of DNA detection period, primer sensitivity and screening of field-collected samples

Feeding trials were conducted in the laboratory to evaluate the detection period, following consumption, of *H*. *variegata* and *A*. *gossypii* DNA in the gut of *C*. *septempunctata* and of *C*. *septempunctata* and *A*. *gossypii* DNA in the gut of *H*. *variegata*. Recently moulted fourth instars of *C*. *septempunctata* and *H*. *variegata* were isolated in 15 ml glass test tubes (1.5 cm diameter), containing a strip of filter paper, closed with cotton wool and arranged horizontally (similarly to ref.^26^). Larvae were starved for 12 h (water provided) to induce a constant level of hunger^61^. Then, larvae were individually transferred into clean glass tubes and allowed to feed on the target prey species for 2 h. After feeding occurred, larvae were provided with an excess of *A*. *fabae*. The individuals that did not consume the food item provided during the 2 h interval were discarded from the experiment. After 0, 1, 2, 3, 4, 8, 12, 18, 24 h (for the evaluation of *H*. *variegata* and *C*. *septempunctata* primers) or after 0, 1, 2, 3, 4, 8, 12, 18, 24, 36 h (for the evaluation of *A*. *gossypii* primers) post-feeding period, samples of individuals were instantly killed and transferred to >95% EtOH and stored at −20 °C until DNA extraction. A sample of 5–6 larvae per time period were analysed. The sensitivity of the primers was tested as reported in ref.^62^, in order to determine the minimal number of DNA molecules needed for successful amplification^63^. Briefly, PCR products were cleaned using PureLink® Quick Gel Extraction Kit (Invitrogen, US) following the instructions of the manufacturer, and the DNA concentration was determined with a NanoDrop 2000 spectrophotometer (Thermo Fisher Scientific, US). Then, the number of double-stranded (DS) fragments was calculated for each target gene based on the molecular weight of the amplicons^62^. Stepwise dilutions were performed (10000–10 DS copies μl^−1^) and standardised samples were then used to determine PCR sensitivity. Finally, samples of 204 larvae (151 *C*. *septempunctata* and 53 *H*. *variegata*) collected in the field were screened in PCR reactions to detect positive amplification of aphids and heterospecific ladybirds from the gut content.

### Data analysis

Binomial generalised linear models (GLMs) were used to analyse feeding trial data, to explore the correlation between larvae positive for the target prey species, DNA and the hours post-feeding. For each predator-prey combination, the T50, i.e. the time points when DNA could be detected in 50% of the fed predators were obtained from the models^22,64^. Differences in the detectability between the predator species were evaluated by means of likelihood ratio test (LRT)^65^.

Comparisons between *C*. *septempunctata* and *H*. *variegata* in terms of observed predation vs. aphids and IGP vs. coccinellids were done by means of binomial generalised linear mixed models (GLMMs), with ladybird predator species as a fixed effect and ladybird larvae nested within sampling units as a random effect. The observed predation frequencies were further corrected as in Greenstone *et al*.^34^. For a given prey, the proportion of positive detection by *H*. *variegata* was substituted into the binomial regression model derived from the peculiar predator-prey feeding trials. The model was then solved to obtain the time since feeding required to reach the observed proportion of positive detections. This value was then used as the explanatory variable in the *C*. *septempunctata* feeding trial model. The returned proportion represented the presumed predation for *H*. *variegata* having the same digestion rate of *C*. *septempunctata*. According to refs^22,23,64^, the corrected frequencies were not statistically evaluated but only used to produce a ranking of the predators.

Monte Carlo simulation or parametric bootstrap approaches were previously used to compare observed vs. expected predation predicted from prior defined hypotheses^26,29,35,66^. Here we adopted a similar approach to test whether observed IGP levels in the field were similar or not to those observed in the laboratory (data from ref.^33^) at a given aphid density.

The field IGP by third- and fourth instars (N = 136 for *C*. *septempunctata* and N = 46 for *H*. *variegata*) was analysed against simulated datasets in which the predicted predation is derived from bioassays conducted in Petri dishes. The specific aim was to test whether observed IGP level in the field was similar or not to that observed in the laboratory at a given aphid density.

In particular, new databases had the same number of individuals, but with a different number of associated positive detections. Data were simulated through a model built with two parts, with the first part that returned a probability that the predation was likely to have occurred and the second part that returned a probability that the predation, once occurred, was likely to be detected. Each sample consisted of a draw from both of these two parts, collectively considered as dependent events. In detail, the first part was a binomial model in which the aphid density, derived from the field data, was used to predict expected IGP. For each IG-predator species, the predicted predation was estimated from logistic models fitted to raw laboratory data that were obtained along 6 hours bioassays (data already published in ref.^33^).

Three models were alternatively used: one for the probability to predate upon similarly aged larvae (3^rd^ or 4^th^ instars), a second one for the probability to predate upon smaller larvae (1^st^ or 2^nd^ instars), finally, another one for the probability to predate upon immobile stages (eggs or pupae). For each sample of each simulated dataset, a random encounter was generated, therefore only one of the three abovementioned models was used to generate a predation probability. The second part of the model was a logistic model for the decay rate data, to account for different DNA detectability between the two species. Observed predation values obtained from the molecular analysis of field-collected larvae were thus compared with the expected predation and 95% confidence interval obtained after 5000 iterations. The probability of getting the observed value from the simulated distribution represents the significance level^67^. All data analysis were performed under R statistical environment^68^.

Some ecological assumptions were made regarding the predation rate of ladybird species in the field and all of them did not considerably bias the results. First, for each ladybird larva (*C*. *septempunctata* or *H*. *variegata*) we assumed that at least one encounter with an immature stage of the other ladybird species happened within the last six hours before the collection. Field surveys confirmed the presence of both species at every sampling units and were conducted during the daytime, when the majority of the larvae are usually very active^7^. Ladybird larvae, in particular *C*. *septempunctata*, can cover high distances (~0.5 cm s^−1^) when searching for prey^69^. Considering the high mobility of ladybird larvae, it was likely that at least one encounter happened.

Second, we assumed no relevant variation in aphid densities within the last 6 hours before each sampling. In other words, we assumed that the aphid densities that were detected just before the collection of the predators were the same densities that might have led to predation. In this respect, there is a huge literature that provides a general consensus on the use of the prey density that has been measured just before the survey as predictor of the likelihood of predation^29,66^.

Third, we assumed that 3^rd^ and 4^th^ instars exhibited a similar behaviour as IG-predators at the different aphid densities. This assumptions was necessary because all the functional response bioassays were conducted using 4^th^ instars as predators and 4^th^ or 2^nd^ instars or eggs as prey. However, only a few number of 3^rd^ instar larvae were screened, therefore the approximation should not relevantly bias our results. Fourth, we assumed that predation was not dependent on the density of the IG-predator or on the density of the IG-prey. The previous literature on IGP^7,11^ particularly strengthens on the importance of the density of the shared prey rather than on the density of the IG-predators and IG-prey^7,11^. This because IGP often happens as a result of nutritive needs caused by a reduction of the EG-prey and the predators’ densities are a consequence of that^11^. Anyhow, we confirmed the presence of the two ladybirds at each samplings. In our simulation, the probability of encountering immobile stages (eggs or pupae) of the IG-prey or lower instars (1^st^ or 2^nd^ instars) or similar-aged instars (3^rd^ and 4^th^ instars) was randomly assigned.

## Electronic supplementary material

Supplementary Information
