# Supplementary material for: Molecular detection of field predation among larvae of two ladybird beetles is partially predicted from laboratory experiments
Source: Sci Rep. 2018 Feb 7;8:2594. doi: 10.1038/s41598-018-20830-2 (PMC5803220; doi:10.1038/s41598-018-20830-2)
Supplement: Supplementary file 1 — Supplementary Information [file 41598_2018_20830_MOESM1_ESM.pdf]

Supplementary Information

Molecular detection of field predation among larvae of two ladybird beetles is partially predicted from laboratory experiments

Gabriele Rondoni[1]*, Saleh Fenjan[1, 2], Valeria Bertoldi[1], Fulvio Ielo[1], Khaled Djelouah[2], Chiaraluce Moretti[1], Roberto Buonaurio[1], Carlo Ricci[1], Eric Conti[1]

[1] Department of Agricultural, Food and Environmental Sciences, University of Perugia, Via Borgo XX Giugno 74, 06121, Perugia (PG), Italy

[2] CIHEAM, Mediterranean Agronomic Institute, Via Ceglie 9, 70010, Valenzano (BA), Italy

*Correspondence addressed to: gabriele.rondoni@unipg.it

Supplementary Table S1: Details of the organisms and GenBank accession numbers of the sequences used in multiple sequence alignment. The accession numbers of the sequences obtained from our field samples are reported in bold.

Organism	GenBank Accession Number
Acyrthosiphon caraganae	EU701270
Acyrthosiphon lactucae	EU701272
Acyrthosiphon macrosiphum	EU701273
Acyrthosiphon malvae	EU701276
Acyrthosiphon pisum	EU701279
Acyrthosiphon purshiae	EU701285
Adalia bipunctata	AJ313068.1
Adalia decempunctata	KM451929.1
Amphorophora agathonica	EU701286
Amphorophora ampullata	EU701288
Amphorophora nr. geranii	EU701291
Amphorophora rubicumberlandi	EU701292
Anoecia fulviabdominalis	EU701293
Aphidinae	EU701752
Aphidius ervi	KT708488.1
Aphidoletes aphidimyza	KJ092340.1
Aphidounguis mali	EU701294
Aphis acaenovinae	EU701295
Aphis asclepiadis	EU701424
Aphis caliginosa	EU701296, EU701297
Aphis ceanothi	EU701298
Aphis clerodendri	EU701407
Aphis clydesmithi	EU701299
Aphis coprosmae	EU701300
Aphis coreopsidis	EU701301, EU701302
Aphis cottieri	EU701304
Aphis craccae	EU701305
Aphis craccivora	EU701312
Aphis crassicauda	EU701315
Aphis decepta	EU701318, EU701317
Aphis equiseticola	EU701319
Aphis fabae	EU701322, EU701330, EU701328, EU701326, EU701324
Aphis farinosa	EU701331, EU701332
Aphis glycines	EU701335

Aphis gossypii	EU701364, EU701357, EU701361, EU701362, EU701356, EU701422, EU701419, EU701418, EU701385, EU701384, EU701386, EU701383, EU701382, EU701381, EU701339, EU701380, EU701379, EU701387, EU701389, EU701378, EU701377, EU701391, EU701375, EU701374, EU701392, EU701367, EU701372, EU701395, EU701370, EU701396, EU701397, EU701371, EU701399, EU701400, EU701368, EU701398, EU701393, EU701369, EU701401, EU701403, EU701402, EU701354, EU701352, EU701351, EU701404, EU701359, EU701350, EU701405, EU701363, EU701340, EU701406, EU701349, EU701341, EU701410, EU701408, EU701342, EU701409, EU701411, EU701343, EU701412, EU701414, EU701347, EU701346, EU701344, EU701345, **MG847520, MG847521, MG847522, MG847523**
Aphis healyi	EU701423
Aphis hederae	EU701321
Aphis helianthi	EU701431
Aphis holodisci	EU701435
Aphis hyperici	EU701437
Aphis ichigo	EU701438
Aphis idaei	EU701440
Aphis illinoisensis	EU701441
Aphis impatientis	EU701442
Aphis lugentis	EU701443, EU701444
Aphis lupini	EU701445
Aphis maculatae	EU701447, EU701446
Aphis manitobensis	EU701448
Aphis middletonii	EU701455
Aphis minima	EU701458
Aphis nasturtii	EU701462
Aphis neilliae	EU701464, EU701466, EU701465
Aphis neogillettei	EU701468, EU701467
Aphis nerii	EU701470, EU701469
Aphis nr. euphorbiae	EU701471
Aphis oenotherae	EU701472
Aphis oestlundi	EU701475, EU701473, EU701474
Aphis oestlundi	KC897405.1
Aphis pomi	EU701479, EU701476, EU701477, EU701478
Aphis rubifolii	EU701481, EU701480
Aphis rumicis	EU701482
Aphis sedi	EU701373

Aphis solanella	EU701327
Aphis spiraecola	EU701503, EU701495, EU701493
Aphis spiraephila	EU701506
Aphis varians	EU701508
Aphis vernoniae	EU701510
Aphthargelia symphoricarpi	EU701514, EU701512
Artemisaphis artemisicola	EU701516
Asiphonaphis pruni	EU701517
Astegopteryx formosana	EU701518
Aulacorthum dorsatum	EU701519, EU701521
Aulacorthum pterinigrum	EU701523
Aulacorthum solani	EU701525, EU701526
Baizongia pistaciae	EU701529
Bemisia tabaci	KJ591608.1
Boernerina variabilis	EU701530
Brachycaudus cardui	EU701531
Brachycaudus helichrysi	EU701532
Brachyunguis tetrapteralis	EU701537
Braggia columbiana	EU701543
Braggia eriogoni	EU701541
Braggia longicauda	EU701542
Braggia urovaneta	EU701544
Brevicoryne brassicae	EU701547
Calaphis flava	EU701548
Cantharis rustica	KM448150.1
Capitophorus elaeagni	EU701550
Capitophorus hippophaes	EU701551
Capitophorus hudsonicus	EU701553
Carolinaia rhois	EU701555
Cavariella theobaldi	EU701563, EU701562
Cepegillettea myricae	EU701568
Cerataphis bambusifoliae	EU701569
Ceratoglyphina styracicola	EU701570
Ceratovacuna japonica	EU701571
Ceruraphis viburnicola	EU701572
Chaetosiphon fragaefolii	EU701574
Chaitophorus populicola	EU701602
Chrysoperla rufilabris	KR148659.1
Cinara anelia	EU701607
Cinara atlantica	EU701609
Cinara coloradensis	EU701610
Cinara fornacula	EU701614

Cinara laricifex	EU701618
Cinara occidentalis	EU701619
Cinara pergandei	EU701621
Cinara watsoni	EU701625
Clydesmithia	EU701626
Coccinella hieroglyphica	KJ963687.1, KJ966585.1
Coccinella magnifica	AJ429494.1, KM449697.1
Coccinella miranda	GU073927.1
Coccinella septempunctata	GU013586.1, KR130888.1, KR130384.1, KP851144.1, KM850872.1, KM850778.1, KM850775.1, KM850528.1, KM850322.1, KM848755.1, KM849570.1, KM847953.1, KM847494.1, KM847561.1, KM847259.1, KM846953.1, KM846933.1, KM846776.1, KM846493.1, KM846369.1, KM843264.1, KM842148.1, KM841529.1, KM846020.1, KM845026.1, KM844743.1, KM844347.1, KM452668.1, KM443837.1, KM439459.1, KJ204125.1, HQ711964.1, EU392414.1, JX508274.1, JX508257.1, JX508192.1, JX508132.1, JX508027.1, JX508008.1, JF296199.1, JF296201.1, JF296181.1, AJ313071.1, KM449469.1, **MG847526, MG847527**
Colopha ulmicola	EU701627
Coloradoa rufomaculata	EU701628
Drepanaphis carolinensis	EU701632
Drepanaphis kanzensis	EU701633
Drepanaphis simpsoni	EU701634
Drepanosiphum platanoidis	EU701635
Dysaphis plantaginea	EU701636
Elatobium abietinum	EU701637
Eomacrosiphon nigromaculosum	EU701638
Episyrphus balteatus	KR260215.1
Ericaphis fimbriata	EU701642, EU701639
Ericaphis gentneri	EU701645
Ericaphis lilii	EU701646
Ericaphis scammelli	EU701647
Ericaphis wakibae	EU701655
Eriosoma crataegi	EU701659
Eriosoma lanigerum	EU701660
Essigella knowltoni	EU701662
Eucallipterus tiliae	EU701665
Eulachnus rileyi	EU701667
Forda marginata	EU701668

Forda riccobonii	EU701669
Frankliniella occidentalis	KR141362.1
Geocoris pallens	KR036760.1
Geoica wertheimae	EU701670
Gharesia polunini	EU701671
Greenidea nipponica	EU701672
Greenidea psidii	EU701673
Grylloprociphilus imbricator	EU701675
Gypsoaphis oestlundi	EU701676
Harmonia dimidiata	EF192096.1
Harmonia quadripunctata	KM447142.1
Hayhurstia atriplicis	EU701680
Helicoverpa armigera	KP253145.1
Henosepilachna elaterii	GU073968.1
Hippodamia convergens	AJ429497.1
Hippodamia variegata	KM449469.1, KM447972.1, KM445423.1, KM439665.1, KM450443.1, KM445899.1, KM444433.1, KM442834.1, KM441730.1, KJ965564.1, KJ964021.1, KJ963952.1, KJ962252.1, KJ961736.1, HQ164778.1, GU073936.1, JQ240197.1, JN580802.1, **MG847524, MG847525**
Hormaphis cornu	EU701682
Hyadaphis tataricae	EU701684, EU701683
Hyalopterus pruni	EU701688, EU701687
Hyperomyzus lactucae	EU701692
Hyperomyzus pallidus	EU701694
Hyperomyzus rhinanthi	EU701695
Liosomaphis berberidis	EU701709
Lipaphis pseudobrassicae	EU701711
Longistigma caryae	EU701716
Lysiphlebus testaceipes	KJ087120.1
Macrosiphoniella leucanthemi	EU701717
Macrosiphoniella ludovicianae	EU701718
Macrosiphoniella millefolii	EU701719
Macrosiphoniella subterranea	EU701720
Macrosiphum albifrons	EU701721
Macrosiphum californicum	EU701722
Macrosiphum coryli	EU701723
Macrosiphum daphnidis	EU701724
Macrosiphum euphorbiae	EU701728, EU701726
Macrosiphum impatientis	EU701731
Macrosiphum parvifolii	EU701732

Macrosiphum rosae	EU701733
Macrosiphum stanleyi	EU701740
Macrosiphum tuberculaceps	EU701742
Maculolachnus sijpkensi	EU701743
Mastopoda pteridis	EU701745, EU701744
Melanaphis bambusae	EU701746
Melaphis rhois	EU701748
Metopeurum fuscoviride	EU701751
Mindarus obliquus	EU701757, EU701758
Mindarus sp.	EU701756
Monaphis antennata	EU701759
Monellia caryella	EU701760
Mordwilkoja vagabunda	EU701761
Muscaphis escherichi	EU701762
Muscaphis musci	EU701763
Muscaphis stroyani	EU701764
Muscaphis utahensis	EU701765
Myzaphis rosarum	EU701766
Myzia oblongoguttata	KM451601.1
Myzocallis asclepiadis	EU701768, EU701767
Myzocallis bellus	EU701769
Myzocallis coryli	EU701770
Myzocallis punctatus	EU701771
Myzodium mimulicola	EU701773
Myzodium modestum	EU701780
Myzus ascalonicus	EU701783, EU701782
Myzus cerasi	EU701788
Myzus lythri	EU701792
Myzus ornatus	EU701794
Myzus persicae	EU701804, EU701800, EU701798, EU701796, EU701795
Nabis punctatus	JQ782830.1
Nasonovia alpina	EU701807, EU701806
Nasonovia aquilegiae	EU701808, EU701809
Nasonovia cynosbati	EU701810
Nasonovia purpurascens	EU701811
Nasonovia ribisnigri	EU701813, EU701812
Nasonovia takala	EU701814
Nearctaphis crataegifoliae	EU701815
Nearctaphis sensoriata	EU701817
Neoamphorophora kalmiae	EU701818
Neomyzus circumflexus	EU701820

Neoprociphilus aceris	EU701822
Nephus includens	GU073951.1
Obtusicauda coweni	EU701823
Oenopia conglobata	KM446481.1
Oestlundiella flava	EU701824
Orius laevigatus	KM016075.1
Pachypappa pseudobyrsa	EU701825
Pachypappa sacculi	EU701828
Paradoxaphis aristoteliae	EU701829
Paradoxaphis plagianthi	EU701830
Peltaphis hottesi	EU701831
Pemphigus betae	EU701832
Pemphigus bursarius	EU701834
Pemphigus monophagus	EU701836
Pemphigus populiramulorum	EU701842
Pemphigus populitransversus	EU701844
Periphyllus lyropictus	EU701850
Periphyllus negundinis	EU701851
Periphyllus testudinaceus	EU701852
Phytoseiulus persimilis	KP642058.1, FM210193.1
Pleotrichophorus pseudoglandulosus	EU701854
Pleotrichophorus xerozoous	EU701855
Plocamaphis flocculosa	EU701856
Prociphilus caryae	EU701857
Prociphilus fraxinifolii	EU701859
Prociphilus tessellatus	EU701863
Prociphilus xylostei	EU701866, EU701868
Propylea japonica	KM207067.1
Pseudoepameibaphis glauca	EU701873
Pterocomma beulahense	EU701876
Pterocomma bicolor	EU701878
Pterocomma sanguiceps	EU701879
Rhopalomyzus lonicerae	EU701881
Rhopalosiphum cerasifoliae	EU701886
Rhopalosiphum insertum	EU701888
Rhopalosiphum maidis	EU701890
Rhopalosiphum nymphaeae	EU701891
Rhopalosiphum padi	EU701894
Rhopalosiphum rufiabdominale	EU701895
Rhyzobius chrysomeloides	KM449740.1
Sanbornia juniperi	EU701896
Sarucallis kahawaluokalani	EU701898

Schizaphis graminum	EU701899
Schizaphis scirpicola	EU701900
Schizolachnus curvispinosus	EU701902
Shivaphis celti	EU701905
Siphonatrophia cupressi	EU701906
Sitobion avenae	EU701907
Spodoptera littoralis	KJ634300.1
Stethorus punctillum	KM849192.1
Subcoccinella vigintiquatuorpunctata	KM450224.1
Symydobius americanus	EU701914
Tamalia coweni	EU701916
Tetranychus urticae	GQ141909.1, KM596708.1
Thecabius populiconduplifolius	EU701922
Thecabius populimonilis	EU701925
Tiliphagus lycoposugus	EU701928
Tinocallis ulmifolii	EU701929
Toxoptera aurantii	EU701930
Toxoptera citricidus	EU701937
Trama rara	EU701939
Trialeurodes vaporariorum	KR345934.1, KR031122.1
Tuberaphis coreana	EU701940
Tuberculatus annulatus	EU701941
Tuberolachnus salignus	EU701942
Uroleucon anomalae	EU701943
Uroleucon erigeronense	EU701944
Uroleucon eupatoricolens	EU701946
Uroleucon nigrotibium	EU701951
Uroleucon taraxaci	EU701953
Utamphorophora commelinensis	EU701954
Wahlgreniella nervata	EU701958
